# Metformin exhibited anticancer activity by lowering cellular cholesterol content in breast cancer cells

**DOI:** 10.1371/journal.pone.0209435

**Published:** 2019-01-09

**Authors:** Ankit Sharma, Shreetama Bandyopadhayaya, Kaushik Chowdhury, Tanu Sharma, Rekha Maheshwari, Amlan Das, Gopal Chakrabarti, Vipin Kumar, Chandi C. Mandal

**Affiliations:** 1 Department of Biochemistry, School of Life Sciences, Central University of Rajasthan, India; 2 Department of Surgery, JLN Hospital, Ajmer, India; 3 Department of Biotechnology, National Institute of Technology, Sikkim, Ravangla, India; 4 Department of Biotechnology and Dr. B.C. Guha Centre for Genetic Engineering and Biotechnology, University of Calcutta, Kolkata, India; 5 Department of Pharmacy, Central University of Rajasthan, India; Roswell Park Cancer Institute, UNITED STATES

## Abstract

Metformin, a widely prescribed anti-diabetic drug, shows anticancer activity in various cancer types. Few studies documented that there was a decreased level of LDL and total cholesterol in blood serum of metformin users. Based on these views, this study aimed to determine if metformin exhibits anticancer activity by alleviating cholesterol level in cancer cells. The present study found that treatment of breast cancer MDA-MB-231 cells with metformin significantly decreased cholesterol content with concomitant inhibition of various cholesterol regulatory genes (e.g., HMGCoR, LDLR and SREBP1). Metformin decreased cell viability, migration and stemness in metastatic MDA-MB-231 cells. Similarly, metformin treatment suppressed expressions of anti-apoptotic genes BCL2 and Bcl-xL, and mesenchymal genes vimentin, N-cadherin, Zeb1 and Zeb2 with simultaneous enhancement of apoptotic caspase 3 and Bax, and epithelial genes E-cadherin and keratin 19 expressions, confirming an inhibitory effect of metformin in tumorigenesis. Similar to metformin, depletion of cholesterol by methyl beta cyclodextrin (MBCD) diminished cell viability, migration, EMT and stemness in breast cancer cells. Moreover, metformin-inhibited cell viability, migration, colony and sphere formations were reversed back by cholesterol treatment. Similarly, cholesterol treatment inverted metformin-reduced several gene expressions (e.g., Bcl-xL, BCL2, Zeb1, vimentin, and BMI-1). Additionally, zymography data demonstrated that cholesterol upregulated metformin-suppressed MMP activity. These findings suggested that metformin revealed anticancer activity by lowering of cholesterol content in breast cancer cells. Thus, this study, for the first time, unravelled this additional mechanism of metformin-mediated anticancer activity.

## Introduction

Cancers are the most complex and complicated diseases where both mutations and epigenetic changes within cancer genome widely differ from one tumor to other. It not only causes a large number of mortality, but also accounts a huge economic burden nationwide. Though, aetiology of tumorigenesis has not yet been established well, however, many intrinsic factors including obesity and hormonal disturbance might positively drive tumorigenesis [1]. Similarly, literature also suggested a positive association of cancer risk and/or mortality with diabetes and high cholesterol [1–3]. Present treatment modalities are quite capable to increase overall survival in cancer patients; however, systemic and off-target toxicity are still the greatest hurdles for the success of cancer therapy. Thus, there is a high demand on the use of relatively non-toxic drugs for cancer treatment.

The commonly prescribed anti-diabetic metformin having relatively fewer toxicity exhibits anticancer potential in many cancer tissues as evidenced by cell culture, animal and clinical studies [4]. Metformin exerts its effect through targeting multiple pathways like activating AMPK and inhibiting mTOR, HER2, and NFκB pathways [5]. Moreover, metformin users have lower serum cholesterol level [6–8]. It had been suggested that cancer cells may have requirement of high cholesterol content by increasing activity and/or expressions of HMG-CoA reductase (HMGCoR), a rate limiting enzyme in cholesterol biosynthesis pathway and low density lipoprotein receptor (LDLR)] involved in cholesterol internalization [9–11]. Many studies also demonstrated a cancer promoting role of sterol regulatory element-binding protein 1 (SREBP1)] which promotes transcription of both HMGCoR and LDLR genes [12, 13]. Recent study documented that cholesterol increased cancer cell migration and invasion in renal carcinoma [14]. Thus, the current research work was mainly focused to examine the effect of metformin on cholesterol content in breast cancer cells, since no studies have yet been conducted to see the influence of metformin treatment on cellular cholesterol level in cancer cells.

Here, we reported that metformin showed a reduction of cellular cholesterol content and cholesterol regulatory molecules (e.g., HMGCoR, LDLR and SREBP1) in metastatic breast cancer MDA-MB-231 cells. It was found that cancer cell viability, migration, epithelial to mesenchymal transition (EMT) and stemness in cancer cells were significantly reduced by metformin treatment. To see the impact of cholesterol on cancer potential, we used cholesterol depleting methyl beta cyclodextrin (MBCD) drug in this study. MBCD exhibited decrease in cell viability, migration, EMT and stemness, similar to metformin. Moreover, exogenous cholesterol treatment reversed back the metformin-mediated anti-tumorigenic activities including cell viability, migration, EMT, stemness and matrix metalloproteinase (MMP) activity in breast cancer cells.

These findings submitted that metformin showed anticancer activity by reducing cholesterol level in breast cancer cells. Thus, this study uncovered this mechanism of metformin-inhibited tumorigenic activity.

## Material and methods

### Materials

TRI Reagent (cat no: T9424), was purchased from Sigma Aldrich. MBCD (cat no: TC227), metformin (cat no: RM10257), and cholesterol (cat no: TC101) were obtained from Himedia (Mumbai, India). Cell culture reagents including fetal bovine serum (FBS) were purchased from Himedia, India. cDNA synthesis kit (AB1453A) and Taq polymerase (MBT060A) were taken from Thermo Scientific (Vilnius, Lithuania) and Himedia, respectively. Cholesterol assay kit (cat no: 71LS200) was purchased from Span diagnostics ltd (Gujarat, India). Antibodies for SREBP1 (cat no: 04–469) and HMGCoR (cat no: MABS1233) from EMD Millipore (CA, USA), vimentin (cat no: CST-5741) from cell signalling technology (MA, USA), BCL2 (cat no: MAB8272) and GAPDH (cat no: MAB5718) from R&D systems (Minnesota, USA), and actin (A02066) from Sigma Aldrich (St. Louis, MO, USA) were used.

### Cell culture

Human breast cancer cell lines MDA-MB-231and MCF-7 were obtained from NCCS, Pune, India. Cells were cultured in DMEM with 10% FBS and 1% pen-strep in 5% CO_2_ level along with humid environment_._

### Cellular and tissue cholesterol extraction

Cancer cells (200x10^3^cells/plate) were seeded in 35 mm plates and subsequently treated with or without test factors. After 24 hrs of treatment, cells were trypsinized and counted. Equal numbers of both treated and untreated cells were used to do cholesterol extraction and measurement. Cholesterol was extracted with chloroform-methanol ratio (2:1) and kept at room temperature to evaporate [15]. After drying, lipid extracts were resuspended in isopropanol to estimate cholesterol. In case of tumor tissues, Equal weights of cancerous tissues (benign and malignant) were obtained and crushed separately in chloroform-methanol ratio (2:1). After crushing, the tissue debris was removed, and the upper liquid was taken out and dried. Colorimetric-cholesterol assay kit was used to measure cholesterol. Absorbance was taken at 505 nm.

Cancer tissues were collected from JLN hospital, Ajmer, Rajasthan, India. The experiments were undertaken with the understanding and consent of each subject. This work was approved by local institutional ethical committee (IEC) at Central University of Rajasthan. This work has been conducted following IEC guideline (Declaration of Helsinki).

### MTT assay

To determine cell viability, cells were seeded in 96 well plates with a density of 5X10^3^ cells/ well and grown up to 70–80% confluence. Cells were then treated with or without test factor for 24 hrs. Next, MTT assay was performed as described earlier [16].

### RNA extraction and RT-PCR

Total RNA from tissues or cells was extracted by TRI reagent as described before [17–19]. In brief, cDNA was prepared from isolated RNA (1 μg) by using Thermo verso cDNA kit. 1 μl of cDNA was used, and PCR reactions were performed by using gene specific primers. PCR products were run on 1.5–1.8% agarose gel and pictures were taken by Gel Doc system. The primers used in this study were given in **S1 Table**.

### Apoptosis assay

Cultured MDA-MB-231 cells were treated grown to a density of 1x10^5^ cells and treated with different concentrations of metformin for 24 h. After treatment, cells were centrifuged at 500Xg for 5 minutes and the pellet was washed with 1X PBS. Cells in the pellet were then suspended in 1X Annexin binding buffer and stained with fluorescein isothiocyanate [FITC]-conjugated annexin V and propidium iodide [PI] for 30 min at room temperature in the dark following the manufacturer’s protocol (TACS Annexin V-FITC Apoptosis Detection Kit, R&D Systems, 4830-01-K) [20]. After incubation cells were analyzed for apoptotic and necrotic population using BD FACSCalibur (BD Biosciences). The normal healthy cells, early apoptosis, late apoptosis and necrotic populations were represented by annexin V-negative/PI-negative population, annexin V-positive/PI-negative, annexin V-positive/PI-positive and annexin-negative/PI-positive cells, respectively. The data were analysed using Cell Quest program from Becton-Dickinson [21].

### Scratch assay

To check cell migration, scratch assay was performed as described earlier [16, 22, 23]. A scratch was made by sterile micropipette tip on the monolayer of MDA-MB-231 cells having 90–100% confluency. Further, treatment was given with different test factors. Migration pictures were taken at various time intervals by inverted microscope (Carl Zeiss). Migration area was calculated by using Image J software.

### Colony formation assay

To perform colony formation assay, cells were plated in a seeding density of 2000 cells/ well in 6 well plates, methodology was described before [16]. After 1 day, cells were treated with or without drugs. After 5 days, cells were washed by PBS and stained with crystal violet. Pictures of both colonies and plates were taken.

### Spheroid formation assay

Spheroid formation assay was performed as described earlier[16], In short, 2X DMEM medium was mixed with 20% FBS and 2% antibiotic pen-strep. Base layer was prepared by adding equal volume of 1.2% agar and 2X DMEM. MDA-MB-231 cells were trypsinized, counted and plated in a density of 1000 cells per plate (35 mm plate). Top layer was prepared by adding equal volume of 0.6% agar and 2X DMEM containing cells. Pictures of spheroids were taken by microscope and counted.

### Western blot analysis

Total cellular proteins were used in western blotting experiments as described before [16, 19, 22, 24]. In brief, total proteins were extracted from treated and untreated cells by using RIPA buffer and estimated by Bradford method. Equal amount of proteins of treated and untreated cells were loaded in SDS-PAGE to resolve proteins, and subsequently transfer proteins from SDS-PAGE to PVDF membrane. Blots were developed with different primary antibodies such as HMGCoR, SREBP1, BCL2, vimentin, GAPDH and actin, and anti-rabbit/mouse secondary antibody. Blot Scanner was used to scan blot.

### Gelatin zymography for MMP activity

Zymography was performed to investigate the MMP activity in metformin-, cholesterol- treated and untreated MDA-MB-231 cells. Protocol was described before [18]. In short, equal volumes of conditioned medium from metformin, metformin plus cholesterol treated and untreated cells were loaded in SDS-PAGE having 5% gelatin. Electrophoresis was performed in non-reducing condition. After electrophoresis, gel was washed with 1.5% TritonX100 in 50 mM Tris buffer at pH 7.5. Next, the gel was incubated with incubation buffer [50 mM Tris (pH 7.5), 200 mM NaCl, 5 mM CaCl_2_, and 5 μM ZnCl_2_] for 24hrs at 37°C. Gel was then stained with coomassie brilliant blue. After distaining the gel, images of gelatinolytic activity (white bands in blue background) were taken.

### Statistical analysis

Statistical analysis was done using Graph pad prism [18, 25, 26]. All values are expressed as mean ± SEM of three replicates. t-test was performed between two different groups. *p<0.05 was considered significant.

## Results

### Higher cholesterol level and cholesterol regulatory gene expressions in malignant breast tissues

**Fig 1:**
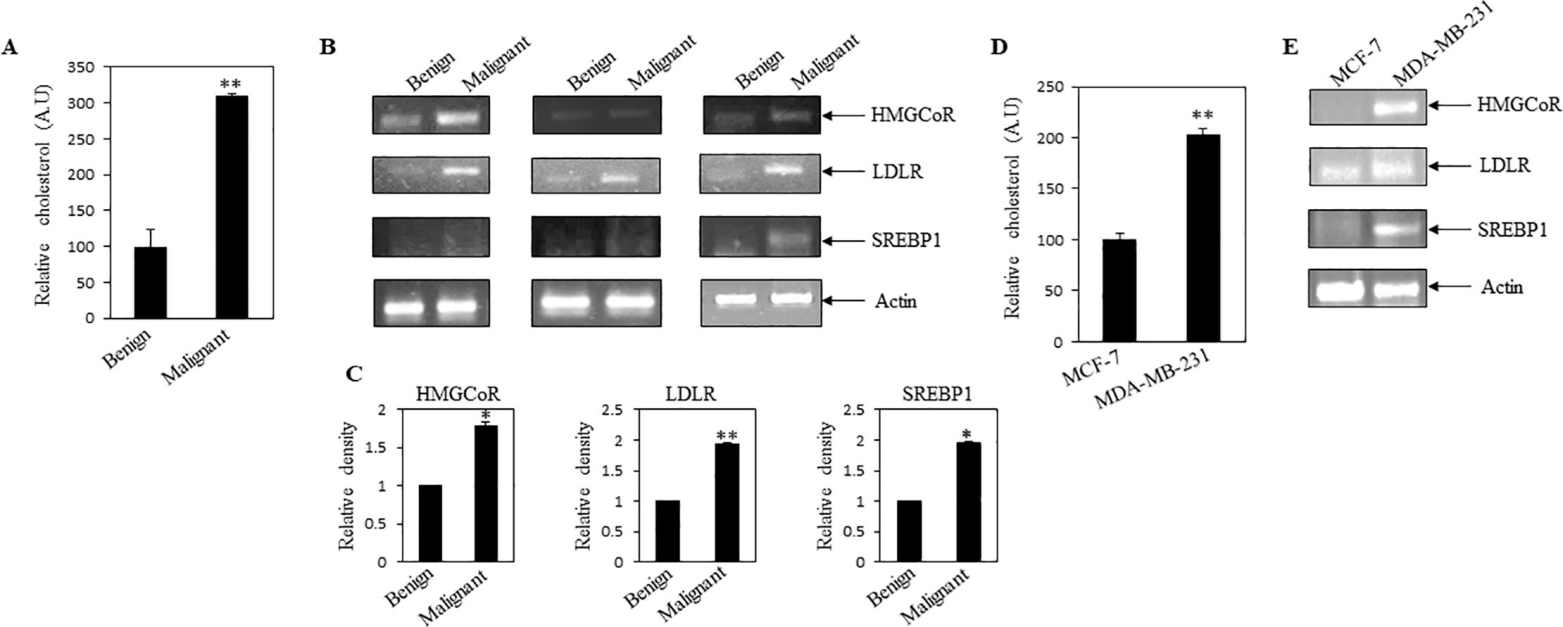
Elevated cellular cholesterol present in malignant breast cancer tissues and metastatic MDA-MB-231 cells. (**A**) Cholesterol level was compared between benign and malignant breast cancer tissues. Equal weights of breast cancer and benign tissues were taken for cellular cholesterol measurement. Bars represent normalized value of cholesterol present in equal weight of tissues. Higher cholesterol content was found in malignant cancer tissues as compared to benign. Values represent mean ± SEM (n = 9). **p < .01 vs. benign. (**B**) RT-PCR was performed by using total RNA of benign and breast cancer tissues with gene specific primers. Expressions of cholesterol regulatory genes were compared. RT-PCR analysis showed higher expressions of HMGCoR, LDLR and SREBP1 genes in malignant cancer tissues. Here, actin was measured as internal control. (**C**) Bars showed densitometry analysis (ratio of concerned gene/ internal loading control). Values represent mean ± SEM (n = 3), *p < .05, **p < .01 vs. benign. (**D**) Cholesterol level was compared in non-metastatic MCF-7 and metastatic MDA-MB-231 cells. Equal numbers of cells from both cell types were taken for cholesterol estimation. Values represent mean ± SEM of triplicate measurements, **p < .01 vs. MCF-7. (**E**) RT-PCR was performed by using equal amount of total RNA isolated from MCF-7 and MDA-MB-231 cells. Here, actin was used as internal control. A.U: arbitrary unit; HMGCoR: HMG-CoA reductase; LDLR: low density lipoprotein receptor; SREBP1: sterol regulatory element-binding protein 1. Benign: fibroadenoma breast tissues (not cancerous); Malignant: malignant breast cancer tissues (as per histological staining).

To know the association between cellular cholesterol and breast cancer malignancy, we compared the cholesterol content present in malignant and benign breast tissues. It was found that malignant breast cancer tissues contained an increased cholesterol level when compared to benign breast tissues (**Fig 1A**). To scrutinise the molecular mechanism of cholesterol enrichment in malignant tissues, expressions of various cholesterol regulatory genes were measured by RT-PCR analysis. These analyses showed higher expressions of cholesterol regulatory gene (i.e., HMGCoR, LDLR and SREBP1) transcripts in malignant tissues as compared to benign breast tissues (**Fig 1B** and **1C**).

According to the previous report, LDL treatment was responsible for the increased proliferation of oestrogen receptor alpha negative (ER-) or triple negative breast cancer (TNBC) cells as compared to oestrogen positive MCF-7 cells, where TNBC contained more cytoplasmic lipid droplets as compared to MCF-7 cells[27]. Also it has been documented that triple negative MDA-MB-231 cells showed elevated cholesterol as compared to non-invasive SU149 breast cancer cells and normal breast MCF10a cells [28]. Elevated expression of LDLR was reported in triple negative breast cancer as compared to ER positive breast cancer [29], which might contribute to the aggressiveness of MDA-MB-231 cells compared ER-positive cells such as MCF-7. Very interestingly we observed that, metastatic breast cancer MDA-MB-231 cells showed an enhanced cholesterol level and expressed high transcript levels of the above cholesterol regulatory genes as compared to non-metastatic breast cancer MCF-7 cells (**Fig 1D** and **1E**). Previous studies also found an enhancement of free cholesterol in MDA-MB-231 breast cancer cells as compared to MCF-7 cells[30]. These observations suggested that elevated cholesterol could have a positive link with cancer malignancy and metastasis.

### Metformin decreased cholesterol content and cholesterol regulatory gene expressions in breast cancer cells

**Fig 2:**
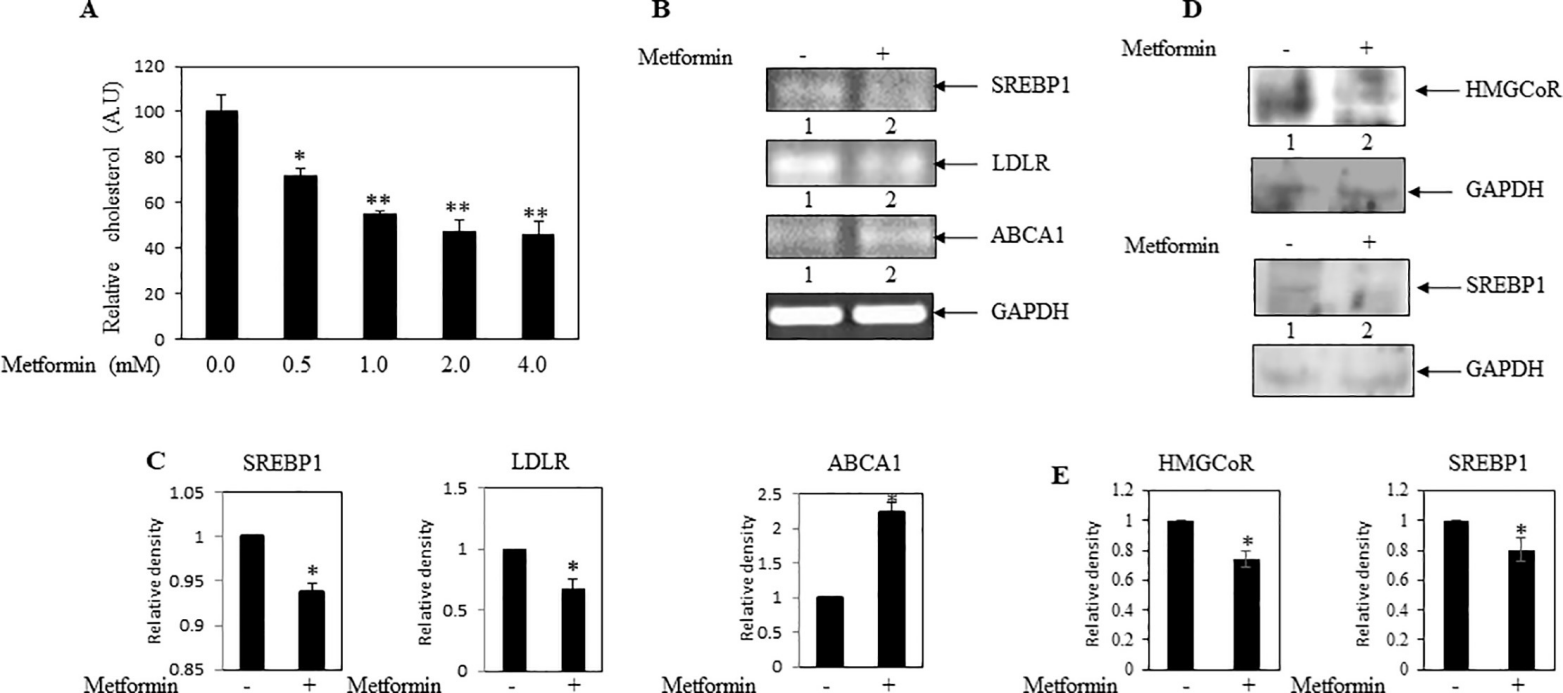
Effect of metformin on cellular cholesterol level and cholesterol regulatory genes in MDA-MB-231 cells. **(A)** MDA-MB-231cells were treated with increasing concentrations of metformin for 24 hrs, and subsequently cholesterol level was estimated in equal number of treated and untreated cells. Bars showed relative cellular cholesterol level. Values represent mean ± SEM of triplicate measurements, *p < .05, **p<01 vs. control. **(B)** RT-PCR analysis was performed using gene specific primers and total RNAs isolated from metformin (2 mM) treated and untreated cells, here GAPDH was used as internal loading control (**C**) Bars showed the densitometry analysis (ratio of concerned gene/ internal loading control). Values represent mean ± SEM of three replicates, *p < .05 vs. control. (**D**) Western blot analysis was performed using total proteins of MDA-MB-231 cells treated with or without metformin (2 mM). (**E**) Bars showed the densitometry analysis (ratio of concerned protein/ internal loading control). Values represent mean ± SEM of three replicates, *p < .05 vs. control. A.U: arbitrary unit; A.U: arbitrary unit; HMGCoR: HMG-CoA reductase; LDLR: low density lipoprotein receptor; SREBP1: sterol regulatory element-binding protein 1; ABCA1: ATP-binding cassette transporter A1.

Some clinical studies submitted that metformin users had a lower serum LDL and total cholesterol level [6–8]. Thus, we intended to determine whether metformin treatment decreases cholesterol level in breast cancer cells. MDA-MB-231 cells were cultured and treated with different concentrations of metformin for 24 hrs. It was found that metformin significantly decreased cholesterol level in a dose dependent manner (**Fig 2A**). Moreover, RT-PCR analyses documented inhibition of SREBP1 and LDLR mRNAs and increment of ABCA1 transcript in metformin treated cells (**Fig 2B** and **2C**). In addition, western blotting analyses exhibited downregulation of both HMGCoR and SREBP1 proteins in metformin treated MDA-MB-231cells as compared to control cells (**Fig 2D** and **2E**). All these data, for the first time, indicated that metformin treatment might inhibit cellular cholesterol level by modulating different cholesterol regulatory molecules (e.g., HMGCoR, LDLR and SREBP1) in breast cancer cells.

### Metformin reduced cell viability in breast cancer cells via apoptosis

**Fig 3:**
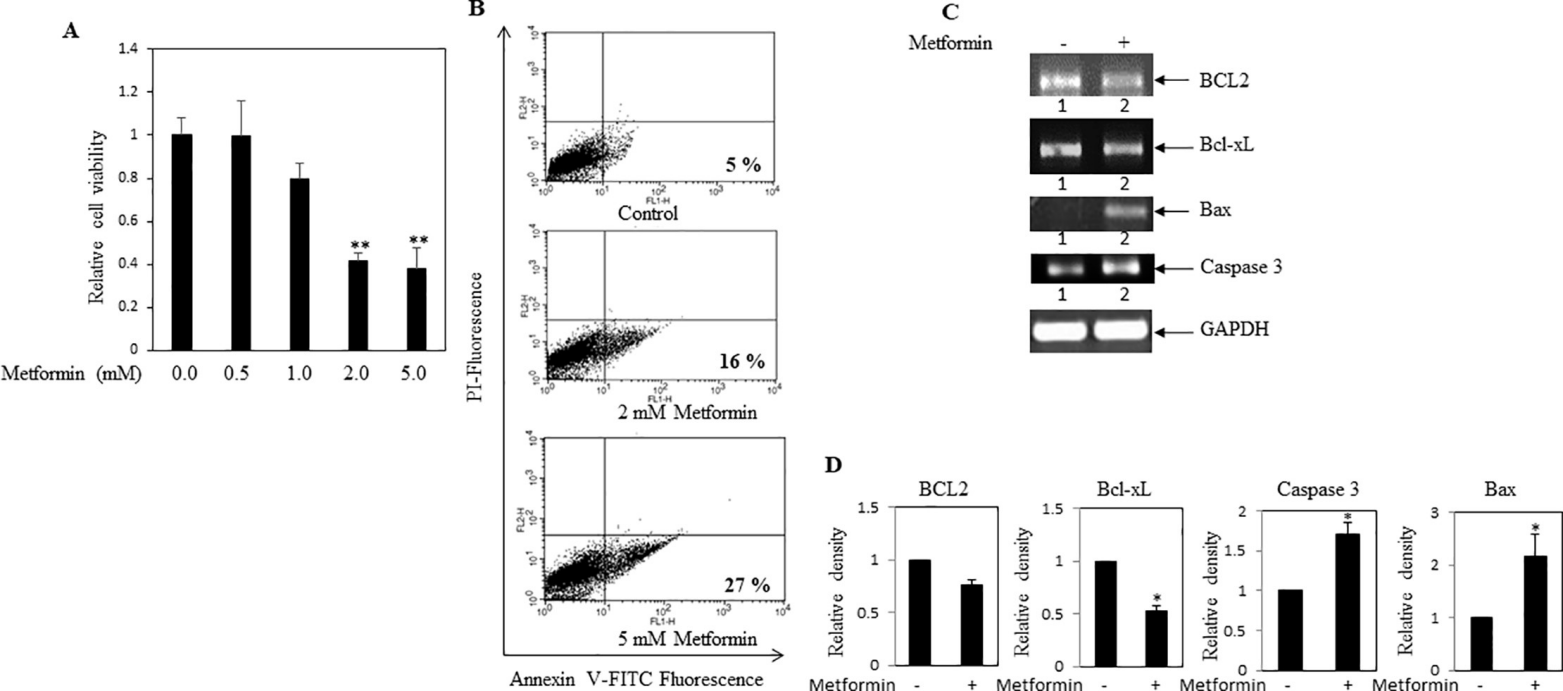
Influence of metformin on MDA-MB-231 cell viability. **(A)** MDA-MB-231 cells were treated with various concentrations of metformin for 24 hrs. MTT assay was performed as an index of cell viability as described in methods. Values represent mean ± SEM of triplicate measurements, **p < .01 vs. control. (**B**) Apoptosis assay was performed as described in methods. Metformin treatment increased the apoptotic cell population as compared to control cells. Here, number of apoptotic cell population was mentioned. (**C)** RT-PCR analysis was performed using total RNA isolated from metformin (2 mM) treated and untreated cells, and gene specific primers. Decreased levels of anti-apoptotic markers BCL2 and Bcl-xL mRNAs were found in metformin treated cells. Elevated levels of apoptotic markers caspase3 and Bax transcripts were observed in metformin treated cells. (**D**) Bars showed densitometry analysis (ratio of concerned gene/ GAPDH). * p < .05 vs. control. Values represent mean ± SEM of three replicates.

Effect of metformin on cell viability was determined. MDA-MB-231 cells were treated with increasing concentrations of metformin. After 24 hrs, MTT assay was performed to calculate viable cell numbers. Numbers of viable cells were dose dependently reduced in metformin treated cells when compared to untreated cells **(Fig 3A)**. To determine the mode of cell death, involvement of apoptosis in metformin treated breast cancer cells was monitored by annexinV-FITC/PI double staining, using flow-cytometry. It was observed that, metformin treatment resulted in a steady increase of apoptotic population in MDA-MB-231 cells in a dose dependent fashion (**Fig 3B**). Moreover, metformin was found to down regulate pro-apoptotic BCL2 and Bcl-xL transcripts, and up-regulated apoptotic Bax and caspase 3 mRNAs (**Fig 3C** and **3D**). Thus, these results indicated that metformin exhibited anticancer activity presumably by augmenting apoptotic activity and diminishing proliferative activity.

### Metformin prevented cell migration and EMT in MDA-MB-231 cells

**Fig 4:**
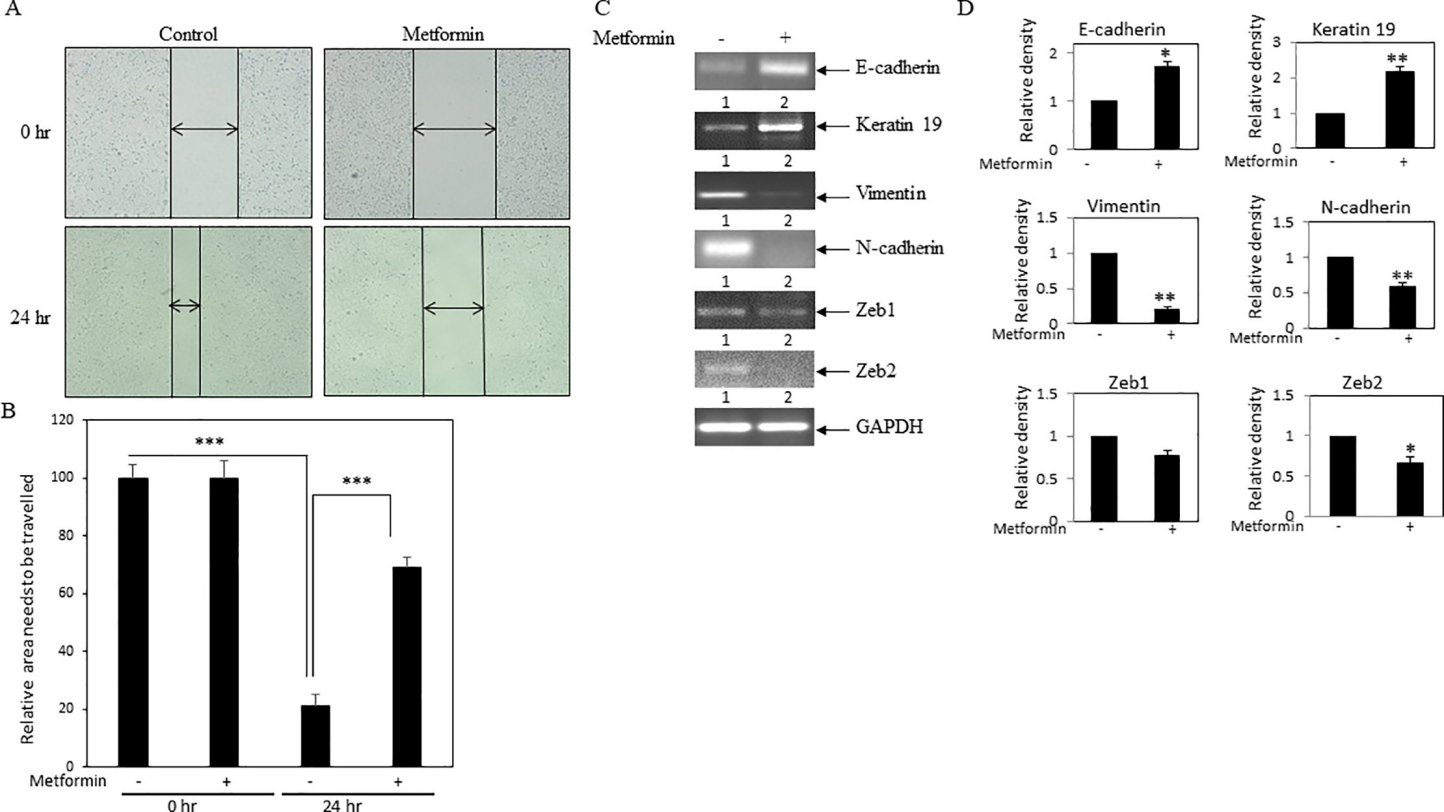
Effect of metformin on breast cancer cell migration and EMT. **(A)** Scratch assay was performed. MDA-MB-231 cell monolayers were scratched and incubated with 2mM metformin for 24 hrs. After the incubation, the cell monolayers were photographed, and represented photos at 0 hr and 24 hr for both control and metformin-treated cells were shown here. **(B)** The unfilled gap areas between two ends of the scratch were measured and subsequently plotted. Significant inhibition of cell migration was observed in case of metformin treated well as compared to control. Values represent mean ± SEM of triplicate measurements, ***p < .001 vs. control at 0 hr and ***p < .001 vs. control at 24 hr. **(C)** RT-PCR analysis was performed using total RNA and gene specific primers. Increased level of epithelial markers E-cadherin and keratin 19 were seen in metformin (2 mM) treated cells. On the other hand, decreased expression of mesenchymal markers vimentin and N-cadherin, and decreased expression of transcription factors Zeb1 and Zeb2 were found in metformin (2mM) treated cells. (**D**) Bars showed densitometry analysis (ratio of concerned gene/ GAPDH), * p < .05 and **p < .01 vs. control.

In this study, to examine the effect of metformin on cancer cell migration, scratch assay was performed. MDA-MB-231 cells were plated and allowed to grow. After reaching 90–100% confluency, a scratch was drawn with a micro tip. Cells were then incubated with or without metformin and were allowed to migrate. After scratching, cells pictures at 0 hr and 24 hr were taken, and representative pictures were shown for both control and treated cells (**Fig 4A**). As expected, cells migrated at 24 hr as compared to 0 hr, since the gap between the two ends of the scratch engineered was reduced in 24 hr pictures as compared to 0 hr pictures. However, the gap between the two ends was larger in case of metformin treated cells when compared to control cells at 24 hr. Moreover, the area in the gap that was to be travelled by cells was calculated by Image J software and plotted (**Fig 4B**). This assay results the confirmed inhibitory effect of metformin on breast cancer cell migration.

EMT is one of the basic steps of metastatic cascade. Moreover, epithelial cancer cells acquire more migratory property when they govern increased mesenchymal phenotype. Thus, we examined whether metformin treatment prevents EMT in breast cancer cells. RT-PCR was performed to check the expressions of various EMT markers. These results showed that metformin treatment increased the expressions of epithelial markers E-cadherin and keratin 19, while decreased the expressions of mesenchymal markers vimentin and N-cadherin, and mesenchymal transcription factors Zeb1 and Zeb2 transcripts (**Fig 4C**). These changes in the gene expressions were confirmed by densitometric analysis (**Fig 4D**).

Thus, these findings proposed that metformin may inhibit cell migration by blocking EMT in breast cancer cells.

### Influence of metformin on stemness in MDA-MB-231 cells

**Fig 5:**
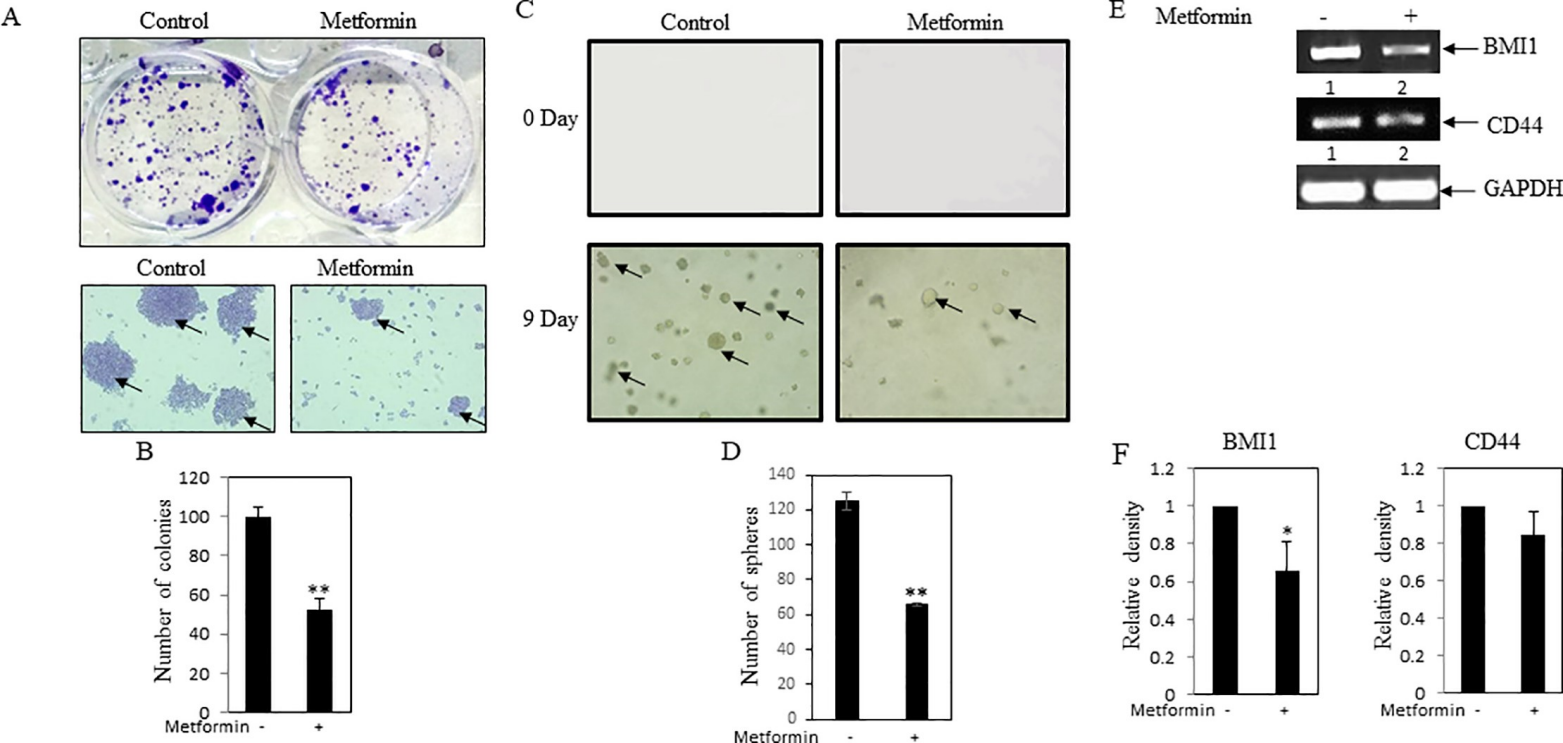
Influence of metformin on stemness property of cancer cells. **(A)** Colony formation assay was performed. MDA-MB-231 cells were plated at low density (1000 cells/well) in 6 well plates. After 24 hrs, cells were treated with metformin (1mM). After 5 days, formed colonies were stained with crystal violet. Photos of the wells were taken by a camera (Upper panel of A). Colonies were visualized by inverted bright field microscope. Arrows depict the colonies. (**B**) Bars represent the average colony count of three independent fields. Value represents mean ± SEM of three replicates, **p < .01 vs. control. (**C**) Soft agar assay of MDA-MB-231 cells in presence and absence of metformin (1mM). Day zero and day 9 pictures were shown in the figure. Arrows depict the spheres. (**D**) Bars showed average of spheroid counts in three independent measurements. Value represents mean ± SEM of three replicates, **p < .01 vs. control. (**E**) RT-PCR analysis was performed using total RNA and gene specific primers. Decreased levels of stemness markers CD44 and BMI1 were found in metformin treated cells. GAPDH was used as internal control. (**F**) Bars showed the densitometry analysis (ratio of concerned gene/ GAPDH), * p < .05 vs. control. Value represents mean ± SEM of three replicates.

Colony formation and soft agar assay were performed to check stemness property of breast cancer cells. It was noticed that number of colonies were significantly reduced in metformin treated MDA-MB-231 cells as compared to control (**Fig 5A** and **5B**). Similarly, soft agar assay documented that when compared to untreated cells, metformin treatment reduced spheroid formations (**Fig 5C** and **5D**). Furthermore, RT-PCR data confirmed inhibition of expressions of stemness marker genes BMI1 and CD44 in metformin treated cells (**Fig 5E** and **5F**). All these data suggested that metformin might inhibit stemness in breast cancer cells.

### Cholesterol depleting MBCD inhibited cancer cell viability, migration and EMT in breast cancer cells

**Fig 6:**
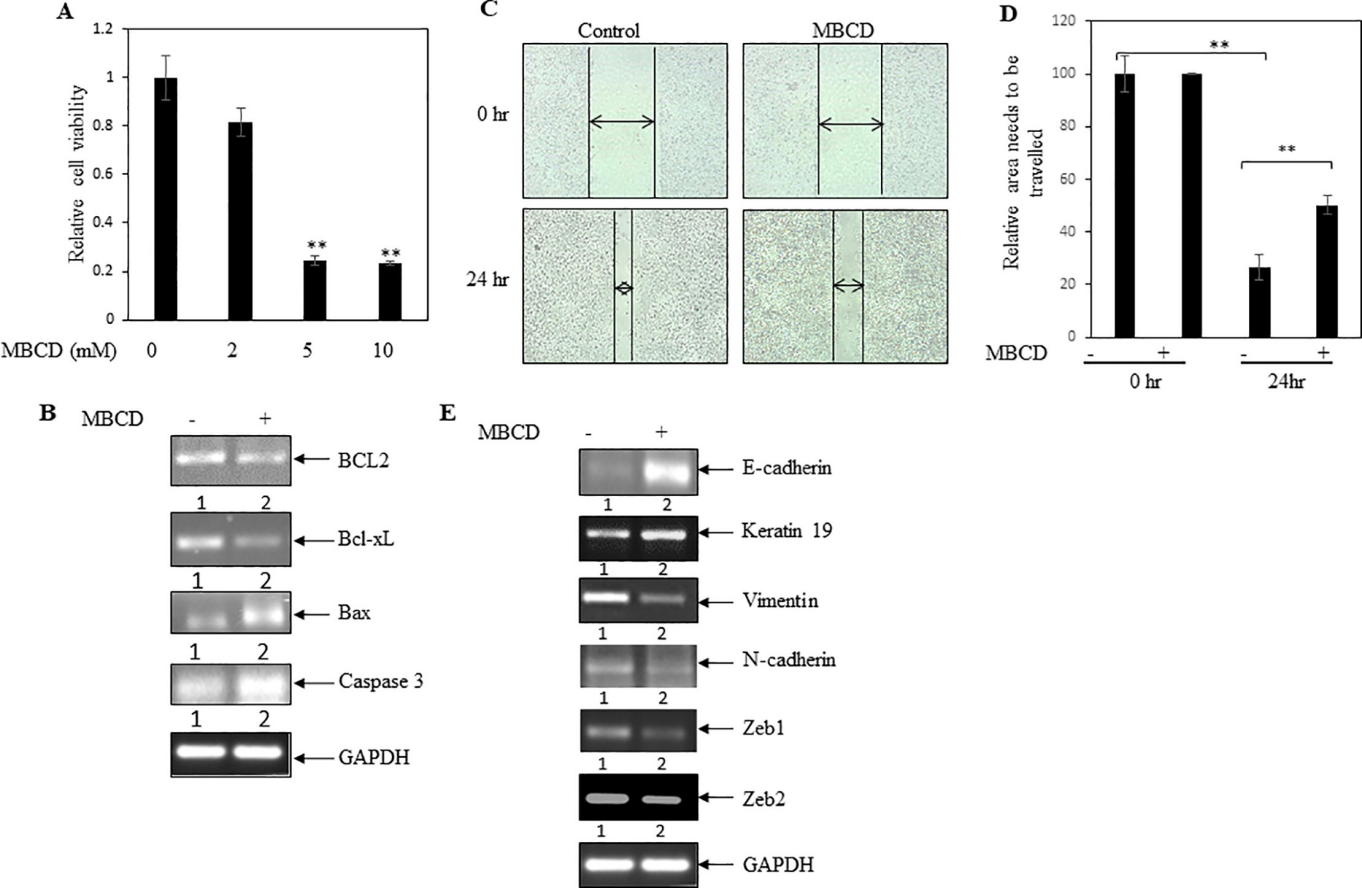
Effect of cholesterol depleting MBCD on breast cancer cells viability and epithelial to mesenchymal transition. (**A**) MTT assay was performed. MDA-MB-231 cells were treated with various concentrations of MBCD for 24 hrs. MBCD inhibited cell viability in a dose dependent manner. Values represent mean ± SEM of triplicate measurements, **p < .01 vs. control. (**B**) RT-PCR analysis was performed using total RNA and gene specific primers. Decreased levels of anti-apoptotic markers BCL2 and Bcl-xL were found in MBCD (4mM) treated cells. On the other hand, elevated level of apoptotic markers caspase 3 and Bax were observed in MBCD treated cells. Densitometry analysis was shown in S1A Fig. (**C**) Scratch assay was performed. MDA-MB-231 cell monolayers were scratched and incubated with MBCD (2mM) for 24 hrs. After the incubation, the cell monolayers were photographed and represented photos at 0 hr and 24 hr for both control and MBCD-treated cells were shown here. (**D**) The unfilled gap areas between two ends of the scratch were measured and subsequently plotted. Significant inhibition of cell migration was observed in case of MBCD treated well as compared to control. Values represent mean ± SEM of triplicate measurements, **p < .01 vs. control at 0 hr. and **p < .01 vs. control at 24 hr. (**E**) RT-PCR analysis was performed using gene specific primers and total RNAs isolated from MBCD treated and untreated cells. Increased levels of epithelial markers E-cadherin and Keratin 19 were seen in MBCD treated cells. On the other hand, decreased expression of mesenchymal markers vimentin and N-cadherin, and decreased expression of transcription factors Zeb1 and Zeb2 were found in MBCD treated cells. Densitometry analysis was shown in S1B Fig.

To determine the influence of cholesterol in cell viability, MDA-MB-231 cells were treated with increasing concentrations of MBCD. After 24 hrs, MTT assay was performed. It was noticed that MBCD treatment significantly blocked cell viability (**Fig 6A**). The above study suggested a positive role of cellular cholesterol in cancer cell viability. However, to investigate the molecular mechanism, we have tested the effect of MBCD on expressions of proliferative marker BCL2 and Bcl-xL mRNAs. Treatment of cancer cells with MBCD showed inhibition of both BCL2 and Bcl-xL transcript levels (**Fig 6B** and **S1A Fig**). Similarly, RT-PCR analysis documented that MBCD significantly increased apoptotic Bax and caspase-3 mRNA levels (**Fig 6B** and **S1A Fig**). Thus, MBCD inhibited cancer cell viability with concomitant reduction of expressions of BCL2 and Bcl-xL, and enhancement of caspase-3 and Bax transcripts.

To test the efficacy of MBCD on cell migration, scratch assay was performed when MDA-MB-231 cells were treated with or without MBCD. Scratch assay documented that MBCD treatment significantly blocked cell migration (**Fig 6C** and **6D**).

Additionally, MBCD treatment increased expressions of epithelial markers E-cadherin and Keratin 19, and decreased expressions of mesenchymal markers vimentin and N-cadherin (**Fig 6E** and **S1B Fig**). Moreover, decreased expressions of mesenchymal transcription factors Zeb1 and Zeb2 were observed in cancer cells treated with MBCD as compared to untreated cells (**Fig 6E** and **S1B Fig**). So, these data indicated that depleting of cellular cholesterol mitigated EMT in breast cancer cells.

### MBCD reduced colony and sphere formation in cancer cells

**Fig 7:**
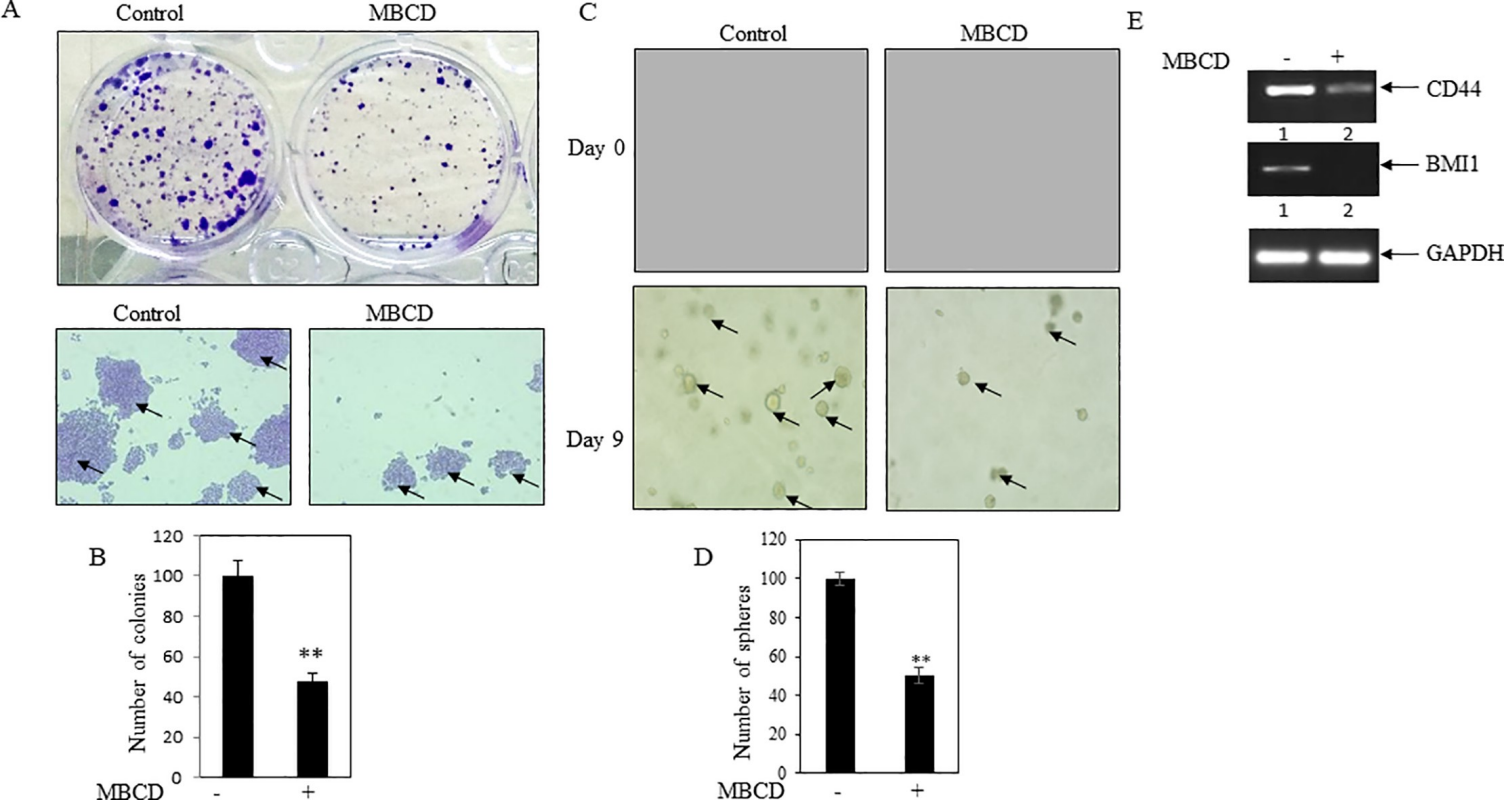
MBCD decreased stemness in MDA-MB-231. (**A**) Colony formation assay was performed. MDA-MB-231 cells were plated at low density (1000 cells/well) in 6 well plates. After 24 hr, cells were treated with MBCD (1mM). After 5 days, formed colonies were stained with crystal violet. Photos of the wells were taken by a camera (Upper panel of A). Colonies were visualized by inverted bright field microscope. Arrows depict the colonies. (**B**) Bars represent the average colony count of three independent fields. Value represents mean ± SEM, **p < .01 vs. control. (**C**) Soft agar assay of MDA-MB-231 cells in presence and absence of MBCD (1mM). Day zero and day 9 pictures were shown in the figure. Arrows depict the spheres. (**D**) Bars showed average of spheroid counts. Value represents mean ± SEM of three independent measurements, **p < .01 vs. control. (**E**) RT-PCR analysis was performed using total RNA and gene specific primers. Decreased levels of stemness markers CD44 and BMI1 were found in MBCD treated cells. GAPDH was measured as loading control. Densitometry analysis was shown in S1C Fig.

To determine the influence of MBCD on the stemness property of cancer cells, colony formation as well as soft agar assay was performed. Data indicated that MBCD significantly decreased colony numbers (**Fig 7A** and **7B**). Simultaneously, soft agar assay revealed that MBCD prevented sphere formation in cancer cells (**Fig 7C** and **7D**). In addition, MBCD decreased the expressions of stemness genes CD44 and BMI 1 (**Fig 7E** and **S1C Fig**). These results suggested that cholesterol depletion inhibited cancer stemness property.

### Cholesterol reversed back metformin-mediated anticancer activity

**Fig 8:**
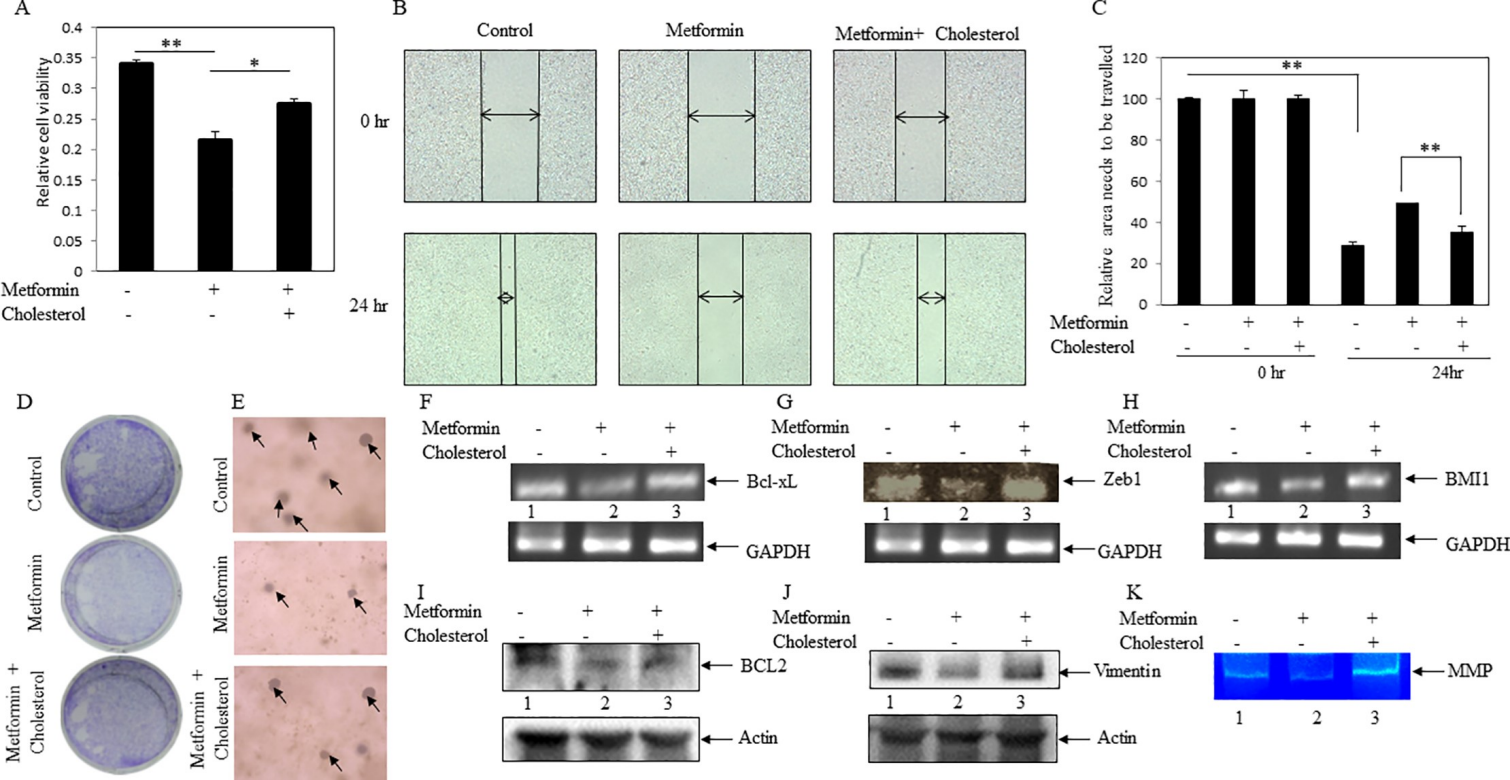
Cholesterol reversed metformin-mediated anticancer potential. Cells were treated with metformin (2 mM) alone and in combination with metformin (2 mM) plus cholesterol (10 μg/ml). (**A**) Cell viability (MTT) assay was performed to check the reverse effect of cholesterol on metformin- inhibited cell viability. Bars are representing the relative cell viability after 24 hr of treatment. Values represent mean ± SEM of triplicate measurements, **p < .001 vs. control and *p < .001 vs. metformin. (**B**) Scratch assay was performed. MDA-MB-231 cell monolayers were scratched and incubated with metformin (2 mM) alone and in combination with metformin (2 mM) plus cholesterol (10 μg/ml). The cell monolayers were photographed, and represented photos at 0 hr and 24 hr for control, metformin and metformin plus cholesterol cells were shown here. (**C**) The unfilled gap areas between two ends of the scratch were measured and subsequently plotted. Values represent mean ± SEM of triplicate measurements, **p < .01 vs. control at 0 hr and **p < .01 vs. metformin at 24 hr. (**D)** Colony formation assay was performed. After 24 hr of cell seeding, cells were treated with metformin (1mM) alone and in combination with metformin (1mM) plus cholesterol (5 μg/ml). After 5 days, formed colonies were stained with crystal violet. Photos of the wells were taken by a camera. (**E**) Soft agar assay of MDA-MB-231 cells in presence and absence of metformin (1mM) alone and in combination with metformin (1mM) plus cholesterol (5 μg/ml). Day 9 pictures were shown in the figure. Arrows depict the spheres. (**F, G** and **H**) RT-PCR analysis was performed using total RNA isolated from metformin, metformin (1mM) plus cholesterol treated and untreated cells, and gene specific primers. Cholesterol treatment reverses the metformin inhibited genes expressions (Bcl-xL [F], Zeb1 [G] and BMI1 [H]) (compare lane 2 vs. lane 3). (**I** and **J**) Western blot analysis was performed using total proteins from metformin, metformin (1mM) plus cholesterol treated and untreated cells, and specific antibodies. Cholesterol treatment reverses the metformin inhibited genes expression (BCL2 [I] and vimentin [J]) (compare lane 2 vs. lane 3). (**K**) Gelatin zymography was performed using equal amount of conditioned medium of metformin, metformin (2 mM) plus cholesterol (10 μg/ml) treated and untreated cells. Metformin-inhibited MMP activity was increased by treatment of cholesterol (compare lane 2 vs. lane 3).

It was described above that both metformin and MBCD inhibited cell viability, migration, EMT and stemness in breast cancer cells. Moreover, metformin reduced cellular cholesterol content in cancer cells. These findings tempted us to investigate whether metformin-mediated anticancer activity is regulated by reducing cholesterol level in the cancer cells. First, we performed MTT assay when cells were either treated with metformin alone or in combination of metformin plus cholesterol. It was observed that cholesterol treatment significantly reversed back metformin-inhibited cancer cell viability (**Fig 8A**). Likewise, migration assay also showed a reversion of metformin-inhibited cell migration in the presence of cholesterol (**Fig 8B** and **8C**). Similarly, both metformin-inhibited colony and sphered formations were rescued in response to cholesterol treatment (**Fig 8D** and **8E**). Moreover, cholesterol treatment inverted metformin-repressed expressions of several cancer-associated genes (e.g., Bcl-xL BCL2, Zeb1, vimentin, BMI1) (**Fig 8F, 8G, 8H, 8I** and 8**J**). It was also noticed in zymography experiment that metformin-reduced MMP activity was inverted by exogenous cholesterol treatment (**Fig 8K**), indicating that cholesterol may induce cancer invasiveness inhibited by metformin. All these findings revealed that cholesterol treatment reversed metformin-mediated anticancer potential. Thus, metformin seems to show anticancer activity by decreasing cellular cholesterol level.

## Discussion

Many investigators including us suggested an existence of positive association between high-cholesterol and cancer risk/mortality [1, 31]. Some contradictory results were also mentioned in the literature [32]. Similarly, substantial data documented that most prescribed cholesterol-lowering statin drugs prevent cancer risk/mortality and tumorigenesis [2, 22, 33]. However, many controversial results sometime limit the application of statins [2, 31]. Thus, finding of statin alternatives is an urgent requirement. This study focused on widely recommended anti-diabetic metformin drug, since many studies documented its anticancer activity in various cancer types [4]. Moreover, few clinical studies also reported a lower level of total cholesterol and LDL cholesterol present in blood serum of metformin users [6–8]. Thus, this study aimed to know whether metformin shows anticancer activity by regulating cellular cholesterol content in cancer cells.

This current study first examined whether breast cancer tissues have elevated cholesterol level. The study of clinical breast tissues exhibited an increased cholesterol level and elevated expressions of few cholesterol regulatory genes (e.g., HMGCoR, LDLR and SREBP1) in malignant breast tumor tissues as compared to benign breast tissues (**Fig 1**). Similarly, metastatic breast cancer MDA-MB-231 cells also showed similar results as compared to non-metastatic MCF-7 cells (**Fig 1**). Other investigators also supported these observations [9–11, 34]. Thus, these findings depicted that elevation of cellular cholesterol might have a cancer promoting role. Our next experimental results demonstrated that metformin inhibited cholesterol level in breast cancer MDA-MB-231 cells with concomitant decrease of cholesterol regulatory gene expressions (e.g., HMGCoR, LDLR and SREBP1) (**Fig 2**). A few studies also showed earlier that metformin might inhibit HMGCoR and SREBP1 in different cell types [35–37]. Thus, our systematic study confirmed the role of metformin in lowering of cellular cholesterol and cholesterol regulatory molecules in breast cancer cells.

Similar to other investigators, our cell culture study further confirmed anticancer activity of metformin, since metformin showed inhibition of cell viability, cell migration, EMT and stemness in breast cancer MDA-MB-231 cells (**Figs 3–5**) [38–41]. Previous study also suggested that metformin showed killing effect on triple negative MDA-MB-231 cells by reducing fatty acid synthase[42]. Thus, these findings suggested that metformin showed anticancer activity with concomitant decrease of cellular cholesterol content and cholesterol regulatory molecules. However, the limitations of this study have been mentioned here. Mammosphere assay, FACS analysis for CD24-/CD44+ population and/or serial dilution xenograft mouse model might be performed to confirm the effect of metformin on stemness in breast cancer cells. Similarly, quantitative real-time PCR (qRT-PCR) would have been a far better assay to compare the relative expression of transcripts between different groups than the semiquantitative RT-PCR.

Few studies also confirmed the role of cholesterol/intracellular lipids in cancer progression and metastasis [14, 43]. We had also used cholesterol depleting MBCD to see cancer promoting role of cholesterol. Our studies found that depletion of cholesterol by MBCD inhibited cancer cell viability, migration, EMT, and stemness property of MDA-MB-231 cells (**Figs 6 and 7**). Few scattered studies suggested earlier a preventive role of MBCD in tumorigenesis in several cancer types [44–46]. However, this methodical study further established that depletion of cellular cholesterol might prevent cancer growth and metastasis. Our cell culture and animal model studies also documented previously that cholesterol lowering simvastatin (a statin family) inhibited breast cancer growth and metastasis, and both MBCD and simvastatin reduced breast cancer induced osteoclast activity [18, 22, 33].

A number of possible mechanisms have been reported for metformin-mediated anticancer activity. Metformin activates AMPK during cellular stress, and this activation of AMPK inhibits energy consuming pathways. Activation of AMPK also restricts use of ATP for metabolic pathways. Since rapidly dividing cancer cells need more amount of energy for metabolic purposes, but activated AMPK put a limit for utilization of energy [36, 47, 48]. Other study showed that metformin inhibits gluconeogenesis via activation of LKB1 (liver kinase B1) and AMPK. It has been also reported that metformin was not able to activate AMPK in absence of LKB1 [49]. Activation of AMPK inhibits acetyl-Co A carboxylase activity which leads to the inhibition of fatty acid synthesis and suppression of SREBP1. On the other hand, activation of LKB1 mediated AMPK pathway further inhibits mTORC1 which is key regulator of cancer cell metabolism and growth [50].

Here, we focused to see the influence of cholesterol in metformin-mediated anticancer activity. It was observed that metformin-inhibited cell viability, migration, colony and spheroid formations were reversed back by exogenous treatment of cholesterol in MDA-MB-231 cells (**Fig 8**). Moreover, cholesterol treatment reversed back of metformin-inhibited proliferative markers (e.g., BCL2 and Bcl-xL), EMT markers (e.g., vimnetin and Zeb1) and stemness marker (e.g., BMI 1) (**Fig 8**). In addition, it also increased metformin-inhibited MMP activity in breast cancer cells (**Fig 8**). These findings suggested that metformin showed anticancer activity by lowering of cholesterol level in breast cancer cells. Thus, this study, for the first time, unravelled this new mechanism of metformin-mediated anti-tumorigenic activity. This study further proposed that metformin may not only reduce the diabetic condition, but also mitigate the diabetes associated cancer mortality by lowering cholesterol level in cancer cells.

## Supporting information

S1 Table Sequence details of human gene specific primers used in PCR. (DOCX)

S1 Fig Densitometric analysis of PCR products. Densitometry analysis (**A**) (ratio of respective gene/GAPDH) of these PCR bands was shown for BCL2, Bcl-xL and caspase 3 in case of MBCD treated samples, (**B**) for E-cadherin, keratin 19, vimentin, N-cadherin, Zeb1 and Zeb2 in case of MBCD treated samples, and (**C**) for CD44 and BMI1 for MBCD treated samples. All the density analyses were normalized with respect to GAPDH (loading control). Value represents mean ± SEM of three replicates. Here, * p<0.05 vs. control.
(TIF)
