# Supplementary material for: Metformin exhibited anticancer activity by lowering cellular cholesterol content in breast cancer cells
Source: PLoS One. 2019 Jan 9;14(1):e0209435. doi: 10.1371/journal.pone.0209435 (PMC6326520; doi:10.1371/journal.pone.0209435)
Supplement: S1 Table — (DOCX) [file pone.0209435.s001.docx]

**S1 Table: Sequence details of human gene specific primers used in PCR.**

| Gene | Forward Primer | Reverse Primer |
| --- | --- | --- |
| E-cadherin | TGCCCAGAAAATGAAAAAGG | GTGTATGTGGCAATGCGTT |
| N-cadherin | CGAATGGATGAAAGACCCATCC | GGAGCCACTGCCTTCATAGTCAA |
| CD44 | CGGACACCATGGACAAGTTT | GAAAGCCTTGCAGAGGTCAG |
| BMI1 | GTCCAAGTTCACAAGACCAGACC | ACAGTCATTGCTGCTGGGCATCG |
| Keratin 19 | GCGGGACAAGATTCTTGGTG | CTTCAGGCCTTCGATCTGCAT |
| BCL2 | GTGAACTGGGGGAGGATTGT | GGAGAAATCAAACAGAGGCC |
| Bcl-xL | ACCCCAGGGACAGCATATCA | TGCGATCCGACTCACCAATA |
| Zeb1 | TGCACTGAGTGTGGAAAAGC | TGGTGATGCTGAAAGAGACG |
| Zeb2 | CGCTTGACATCACTGAAGGA | CTTGCCACACTCTGTGCATT |
| Vimentin | TCCAGCAGCTTCCTGTAGGT | CCCTCACCTGTGAAGTGGAT |
| ABCA1 | GCACTGAGGAAGATGCTGAAA | AGTTCCTGGAAGGTCTTGTTCAC |
| SREBP1 | GCAAGGCCATCGACTACATT | GGTCAGTGTGTCCTCCACCT |
| LDLR | CCTGCTAGAAACCTCACATTG | GGATCACGACCTGCTGTGTC |
| GAPDH | GGGAAGGTGAAGGTCGGAG | GAGGGGGCAGAGATGATGA |
| Actin | AGCACTGTGTTGGCGTACAG | AGAGCTACGAGCTGCCTGAC |
