# Supplementary figures and images for: Metformin exhibited anticancer activity by lowering cellular cholesterol content in breast cancer cells
Source: PLoS One. 2019 Jan 9;14(1):e0209435. doi: 10.1371/journal.pone.0209435 (PMC6326520; doi:10.1371/journal.pone.0209435)

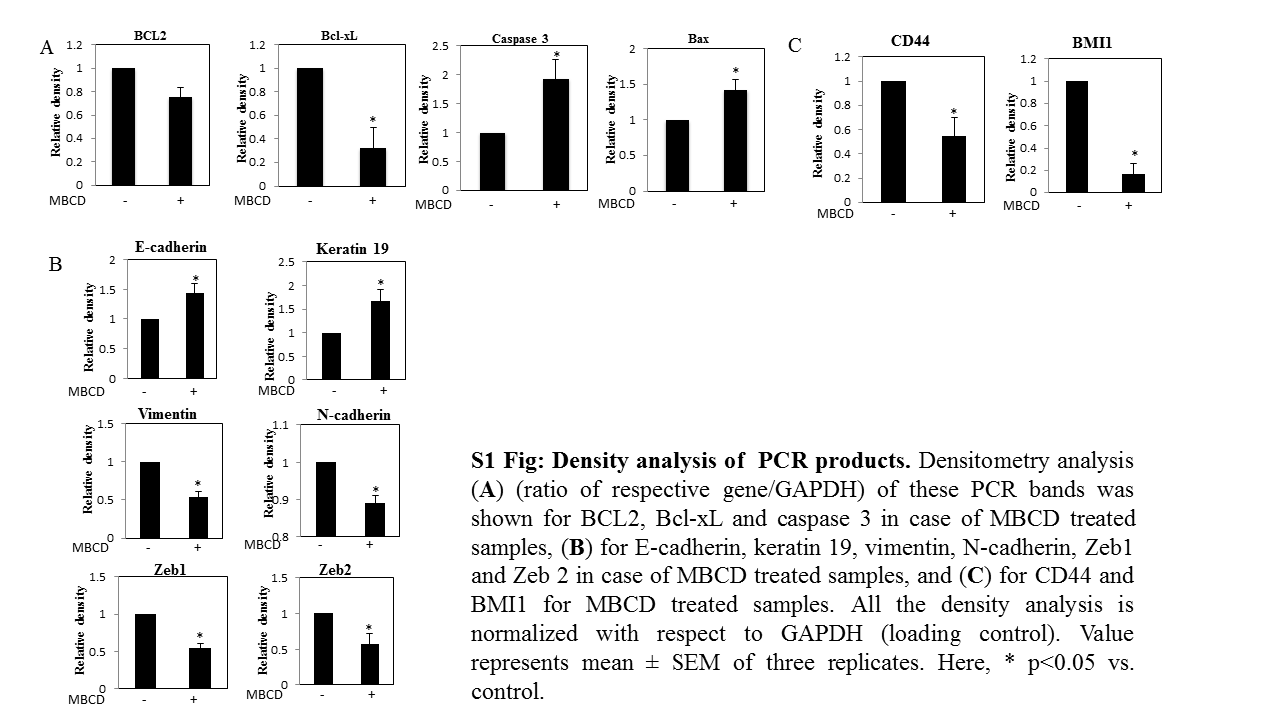

Supplement: S1 Fig — Densitometry analysis (A) (ratio of respective gene/GAPDH) of these PCR bands was shown for BCL2, Bcl-xL and caspase 3 in case of MBCD treated samples, (B) for E-cadherin, keratin 19, vimentin, N-cadherin, Zeb1 and Zeb2 in case of MBCD treated samples, and (C) for CD44 and BMI1 for MBCD treated samples. All the density analyses were normalized with respect to GAPDH (loading control). Value represents mean ± SEM of three replicates. Here, * p<0.05 vs. control. (TIF) [file pone.0209435.s002.tif]
